# A new species of *Xenoturbella* from the western Pacific Ocean and the evolution of *Xenoturbella*

**DOI:** 10.1186/s12862-017-1080-2

**Published:** 2017-12-18

**Authors:** Hiroaki Nakano, Hideyuki Miyazawa, Akiteru Maeno, Toshihiko Shiroishi, Keiichi Kakui, Ryo Koyanagi, Miyuki Kanda, Noriyuki Satoh, Akihito Omori, Hisanori Kohtsuka

**Affiliations:** 1 Shimoda Marine Research Center, University of Tsukuba, 5-10-1, Shimoda, Shizuoka, 415-0025 Japan; 2 Mammalian Genetics Laboratory, National Institute of Genetics, 1111 Yata, Mishima, Shizuoka, 411-8540 Japan; 3 Faculty of Science, Hokkaido University, N10 W8, Kita-ku, Sapporo, Hokkaido 060-0810 Japan; 4 DNA Sequencing Section, Okinawa Institute of Science and Technology Graduate University, Onna, Okinawa, 904-0495 Japan; 5 Marine Genomics Unit, Okinawa Institute of Science and Technology Graduate University, Onna, Okinawa, 904-0495 Japan; 6 Misaki Marine Biological Station, The University of Tokyo, 1024 Koajiro, Misaki, Miura, Kanagawa 238-0225 Japan; 7 Present address: Sado Marine Biological Station, Faculty of Science, Niigata University, Sado, Niigata, 952-2135 Japan

**Keywords:** *Xenoturbella*, Acoels, Nemertodermatids, Acoelomorpha, Xenacoelomorpha, Frontal organ, Deuterostomes, Bilaterians, Metazoans, Evolution

## Abstract

**Background:**

*Xenoturbella* is a group of marine benthic animals lacking an anus and a centralized nervous system. Molecular phylogenetic analyses group the animal together with the Acoelomorpha, forming the Xenacoelomorpha. This group has been suggested to be either a sister group to the Nephrozoa or a deuterostome, and therefore it may provide important insights into origins of bilaterian traits such as an anus, the nephron, feeding larvae and centralized nervous systems. However, only five *Xenoturbella* species have been reported and the evolutionary history of xenoturbellids and Xenacoelomorpha remains obscure.

**Results:**

Here we describe a new *Xenoturbella* species from the western Pacific Ocean, and report a new xenoturbellid structure - the frontal pore. Non-destructive microCT was used to investigate the internal morphology of this soft-bodied animal. This revealed the presence of a frontal pore that is continuous with the ventral glandular network and which exhibits similarities with the frontal organ in acoelomorphs.

**Conclusions:**

Our results suggest that large size, oval mouth, frontal pore and ventral glandular network may be ancestral features for *Xenoturbella.* Further studies will clarify the evolutionary relationship of the frontal pore and ventral glandular network of xenoturbellids and the acoelomorph frontal organ. One of the habitats of the newly identified species is easily accessible from a marine station and so this species promises to be valuable for research on bilaterian and deuterostome evolution.

**Electronic supplementary material:**

The online version of this article (10.1186/s12862-017-1080-2) contains supplementary material, which is available to authorized users.

## Background

*Xenoturbella* is a group of marine benthic worms, first described in 1949 as a ‘strange’ platyhelminth [1]. It has a mouth but lacks an anus, hence the digestive organ is a sack rather than a tube. The nervous system of *Xenoturbella* is not centralized and is in the form of an intraepidermal nerve net [1–3]. Structures such as a coelom and reproductive organs are absent [1]. Various phylogenetic positions have been suggested for this animal based on different morphological characters - an early metazoan group based on its overall body plan [4], the sister group to Bilateria based on its nervous system structure [2] and musculature [5], a member of the deuterostomes based on epidermal structure [6, 7] and a bivalve based on oocyte characteristics [8] (discussed in [9–11]). Recent molecular phylogenetic analyses support a close affinity with the Acoelomorpha [12–14], a group of marine worms also originally suggested to belong to the Platyhelminthes, but later suggested to be the sister group to the Nephrozoa (all remaining Bilateria) [15–21]. *Xenoturbella* and Acoelomorpha are suggested to form a new clade, the Xenacoelomorpha, and accordingly similarities in overall body plan [1], morphology of the free-swimming stage during development [22–24], ciliary ultrastructure [25–27] and degenerating epidermal cells [28] have been reported*.* However, diversity within the Xenacoelomorpha has been reported for some other features, such as the morphology of the digestive organ [1], statocyst [29, 30], sperm [31–33] and cleavage pattern [11, 34–36]. When a monophyletic clade consisting of *Xenoturbella* and Acoelomorpha was first proposed based on large scale molecular phylogenetic analyses, it was suggested to be a sister group to the Nephrozoa [12]. A later study, in which the name Xenacoelomorpha was first introduced, proposed that the group is a member of the deuterostomes [13]. However, a more recent study has again suggested a sister group relationship to the Nephrozoa [14]. Either way, it is clear that studies on *Xenoturbella* could provide important insights into the origins of bilaterian traits such as an anus, the nephron, feeding larvae and centralized nervous systems [24, 37–43].

The type species for *Xenoturbella*, *X. bocki*, is about 1–3 cm in body length and inhabits the seafloor of western Sweden coast at 50–200 m depth [1, 9, 11]. There is a considerable body of research on this species, and almost all knowledge of *Xenoturbella* comes from this animal. A second species, *X. westbladi*, was reported from the same habitat as *X. bocki* in 1999 [44], but a recent haplotype network analysis using cytochrome c oxidase subunit I (*cox1*) sequences showed that *X. bocki* and *X. westbladi* are a single species and that *X. westbladi* should be regarded as a junior synonym for the species [45]. Xenoturbellids were then reported from the Pacific Ocean when four species (*X. monstrosa*, *X. churro*, *X. profunda* and *X. hollandorum*) were reported from the west coast of USA and Mexico in 2016 [45]. This discovery revealed unknown diversity within the group: the body lengths of three of the species were over 10 cm, with *X. monstrosa* reaching about 20 cm; a structure called the ventral glandular network was described; and two sub-clades termed ‘shallow’ and ‘deep’ were identified. These four species all live on the sea floor at over 600 m deep, with some as deep as 3700 m, and require remotely operated vehicles (ROVs) equipped with a slurp gun for collection, making them difficult to work on as research organisms. Here we report the discovery of a new species of *Xenoturbella* off the Japanese coast and discuss the ancestral traits of *Xenoturbella* and Xenacoelomorpha. The new species can be collected using a marine biological dredge within an hour from a marine station, and therefore it promises to be a valuable model for further research on xenacoelomorphs.

## Results

**Xenoturbellida Bourlat et al., 2006** [46]

**Genus** ***Xenoturbella*** **Westblad, 1949** [1]

***Xenoturbella japonica*** **sp. nov.**

**Etymology.** Named for the locality where the specimens were collected.

**Fig. 1:**
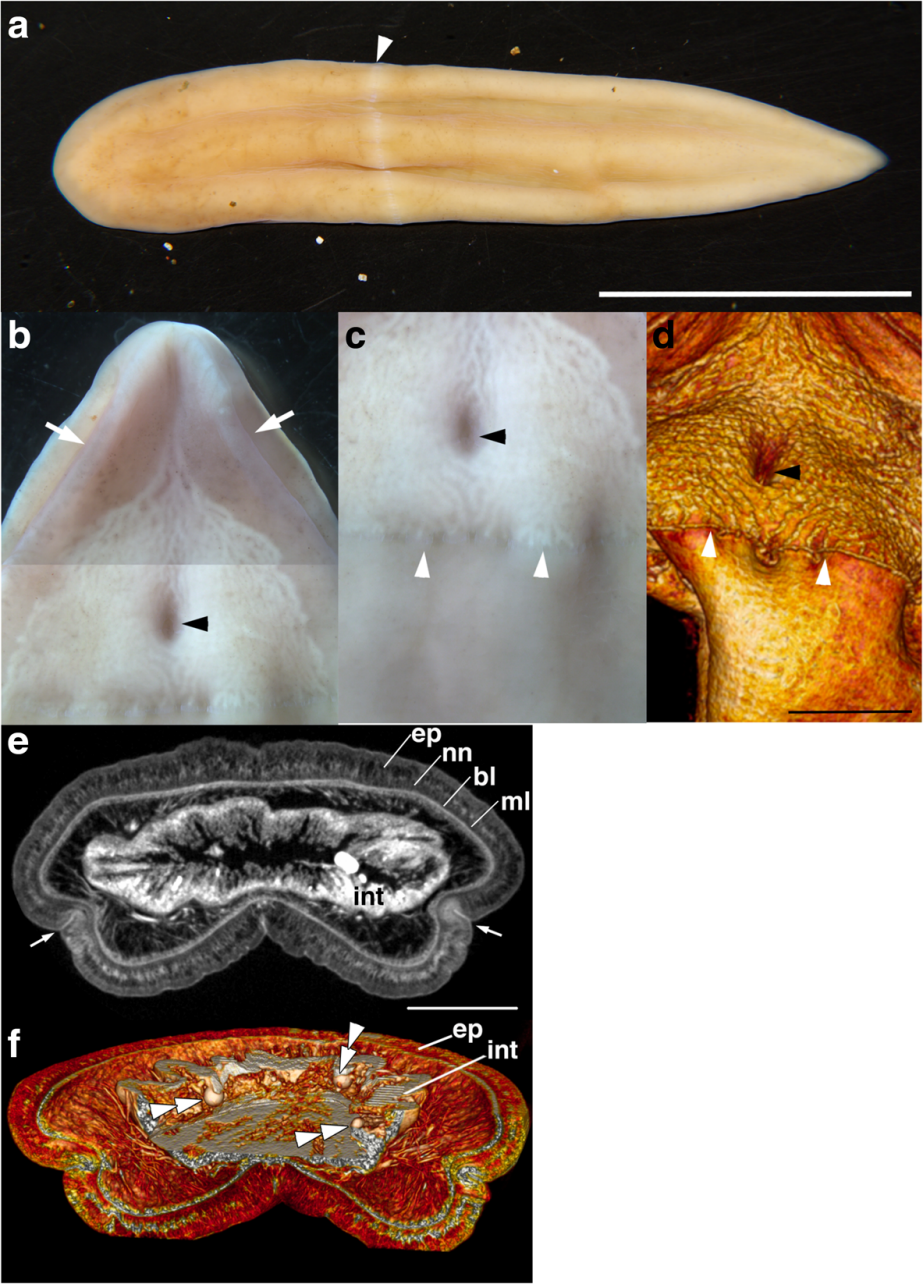
Morphology of the holotype specimen (female) of *Xenoturbella japonica* sp. nov. **a** Live specimen with anterior to the left. **b** Antero-ventral part of a relaxed specimen, composed from two separate photographs. **c** Mid-ventral part of a relaxed specimen. The ventral glandular network ends at the ring furrow. **d** Volume rendering image from microCT scans showing the difference in epidermal composition between the anterior and posterior parts of the animal. **e** MicroCT scan showing a transverse section just anterior to the mouth. The epidermis (ep), intraepidermal nerve net (nn), basal lamina (bl) and muscle layer (ml) surround the intestine (int). **f** Volume rendering image from microCT scans showing oocytes inside the intestine (int). White arrowheads, ring furrow; white arrows, side furrow; black arrowheads; mouth, white double arrowheads, oocytes. Scale bars: a: 2 cm, d: 3 mm, e: 1 mm

**Fig. 2:**
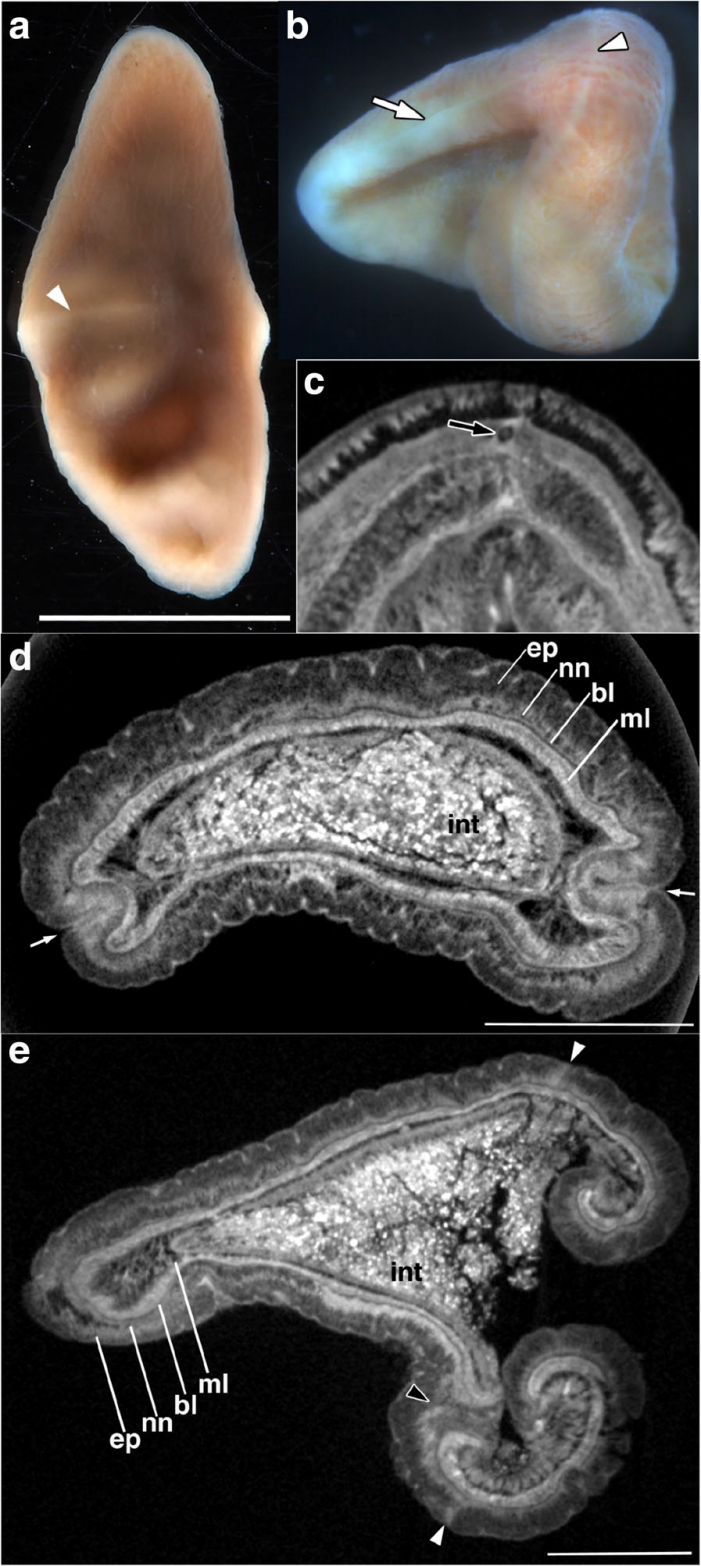
Morphology of the paratype specimen (juvenile) of *Xenoturbella japonica* sp. nov. **a** Live specimen with anterior to the top. **b** Left-ventral view of a contracted specimen after fixation with anterior to the left. **c**–**e** MicroCT scans showing internal morphology. **c** Latitudinal section with anterior to the top. **d** Transverse section just anterior to the mouth. The epidermis (ep), intraepidermal nerve net (nn), basal lamina (bl) and muscle layer (ml) surround the intestine (int). **e** Longitudinal section with anterior to the left. The posterior epidermis, to the right, is curled due to contraction following fixation. White arrowheads, ring furrow; white arrows, side furrow; black arrow, statocyst; black arrowhead, mouth. Scale bars: a: 5 mm, d,e: 1 mm

**Fig. 3:**
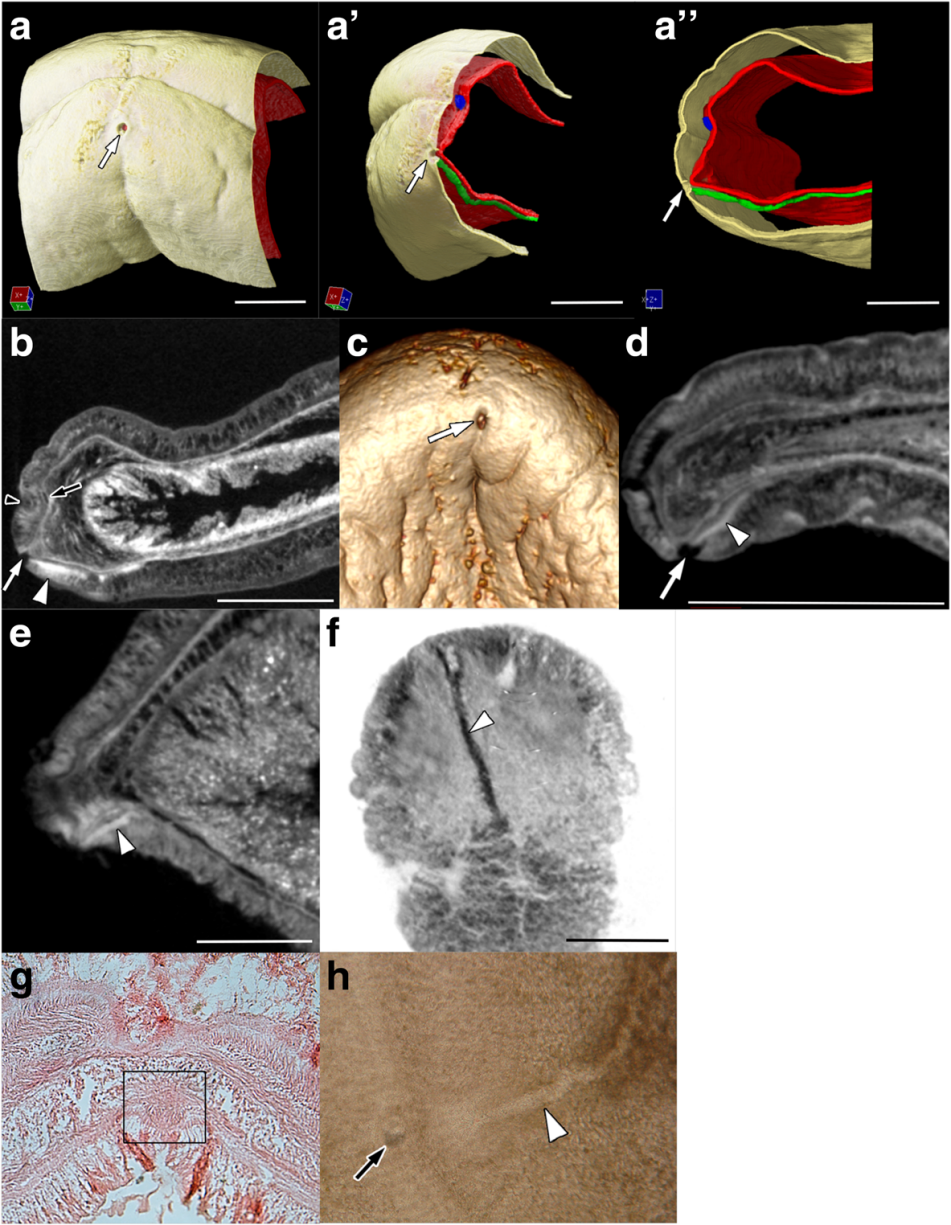
Frontal pore and the ventral glandular network of *Xenoturbella*. a–b: *X. japonica* sp. nov., holotype female specimen. a–a”: Reconstructed images showing the relative positions of the frontal pore, statocyst (blue), ventral glandular network (green) and the basal lamina (red). b: Longitudinal section with anterior to the left. c–d: *X. japonica* sp. nov., paratype juvenile specimen. c: Volume rendering image of the anteroventral tip. d: Longitudinal section with anterior to the left. e–h: *X. bocki*. e: Longitudinal section with anterior to the left. f: Latitudinal section with anterior to the top. g: Transverse section of the anterior part of an animal, with dorsal to the top. The frontal organ is present in the mid-ventral region (black square). h: Anterior part of a specimen pressed under a cover glass. White arrowheads, ventral glandular network; white arrows, frontal pore; black arrowhead, side furrow; black arrows, statocyst. Scale bars: a–a”: 600 μm, b,d: 1 mm, e,f: 500 μm

**Material examined.** Holotype: NSMT-Xe 2, female (Figs. 1, 3, Additional file 1: Video S1 and Additional file 2: Video S2). Paratype: NSMT-Xe 1, juvenile, sex unknown (Figs. 2, 3, Additional file 1: Video S1 and Additional file 3: Video S3).

**Locality**. Holotype: off Jogashima, Miura, Kanagawa, Japan, 35°06.93″ N 139°33.72″ E to 35°06.95″ N 139°33.33″ E; 380–554 m depth (Additional file 4: Figure S1). Specimen found from sediment obtained using a marine biology dredge (Rigo Co., Ltd., Tokyo, Japan) on December 9th, 2015 during a research survey (December 9th to 10th, 2015; JAMBIO application number 27–7) headed by Dr. Hiroshi Namikawa, National Museum of Nature and Science, using the RV *Rinkai-Maru* of the Misaki Biological Marine Station, The University of Tokyo.

Paratype: Sanriku coast, Iwate, Japan, 39°37.86″ N 142°18.22″ E to 39°37.00″ N 142°17.60″ E; 517–560 m depth (Additional file 4: Figure S1). Specimen found inside the inner small plankton net with a mesh size of 0.5 mm attached inside a larger beam trawl [47]. Collected on July 18th, 2013 during a research cruise (July 18th to 29th, 2013) headed by Dr. Ken Fujimoto, National Research Institute of Fisheries Science, Fisheries Research Agency, aboard FRV *Soyo-Maru* of the National Research Institute of Fisheries Science, Fisheries Research Agency, Japan.

**Description of female.** Based on holotype. Body 5.3 cm in length; pale orange with coloration getting darker toward the anterior (Fig. 1a). In live specimens, muscles hold the dorsal body wall in a W-shape (three ridges and two troughs). Body shape actively changes by contracting and elongating when alive. Ring furrow and side furrow are present (Fig. 1a,b). Ventral mouth present, oval-shaped, just anterior to ring furrow (Fig. 1b,c). Glandular network present over ventral surface, starting near anterior tip of body and ending just in front of ring furrow (Fig. 1b–d). Internally, body wall with epidermis, circular and longitudinal muscles, parenchyma and gastrodermis present (Fig. 1e). Oocytes present within intestine (Fig. 1f). Statocyst situated near anterior tip of body, just inside side furrow (Fig. 3a,b).

**Description of juvenile.** Based on paratype. Similar to female, but differs as follows: body 1.1 cm in length; pale orange in color (Fig. 2a); dorsal body surface in live specimen smooth, lacking longitudinal ridges and troughs, similar to that of *X. bocki*; gametes not observed. Ventral glandular network not detected externally, but observed with microCT imaging (Figs. 2 and 3).

**Genetic information.** Whole mitochondrial genome sequences (15,244 bp in holotype; 15,249 bp in paratype) and partial Histone H3 gene sequences (346 bp in holotype; 413 bp in paratype) were determined and deposited as INSD accession numbers LC228486, LC228485, LC228579 and LC228578, respectively. Exogenous mitochondrial and rSSU sequences of the following bivalves were detected: *Acila castrensis* from the holotype; *Nucula nucleus*, *Ennucula cardara*, *A. castrensis* and *Limaria fragilis* from the paratype.

### Frontal pore and ventral glandular network of *X. japonica* and *X. bocki*

Volume rendering imaging of the anterior end of the holotype *X. japonica* specimen revealed that a frontal pore was present at the anterior tip of the body, ventral to side furrow (Fig. 3a). MicroCT imaging confirmed the presence of the pore, and also showed that the ventral glandular network continued posteriorly from the pore while branching (Fig. 3a,b, Additional file 2: Video S2). Analyses of the paratype juvenile specimen also showed that a frontal pore was present, and that the ventral glandular network, a linear structure, continued posteriorly from the pore along the basal lamina (Fig. 3c,d, Additional file 3: Video S3). Based on the discovery of these structures in the new species, we decided to reinvestigate the type species of *Xenoturbella*, *X. bocki*. MicroCT imaging showed that the frontal pore and the ventral glandular network are also both present in *X. bocki* (Fig. 3e,f, Additional file 1: Video S1 and Additional file 3: Video S3). Histological sections and light microscopic observations confirmed the presence of the structure (Fig. 3g,h).

### Molecular phylogenetic analyses

The mitochondrial genome sizes of the *X. japonica* holotype and paratype were 15,244 bp and 15,249 bp, respectively. The gene content and gene order were identical with those of other *Xenoturbella* species (Additional file 5: Figure S2) [45, 48, 49].

**Fig. 4:**
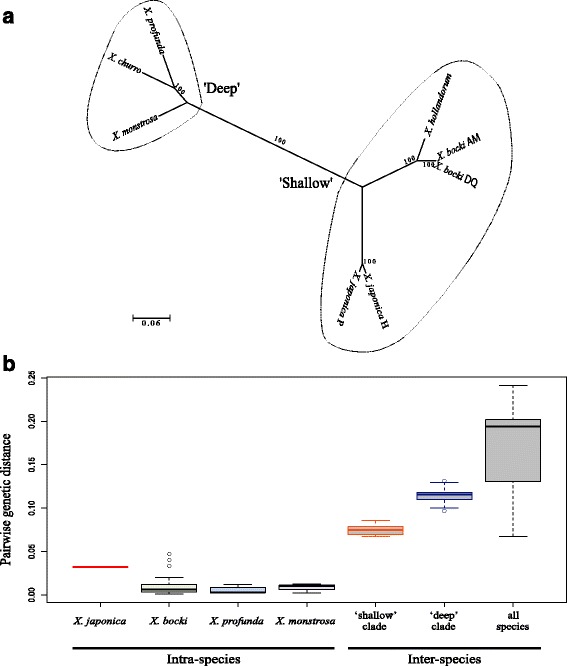
Internal phylogeny of *Xenoturbella*. **a** Unrooted maximum likelihood tree of *Xenoturbella* species based on mitochondrial 13 protein-coding genes. Bootstrap values are shown at the nodes. H: holotype, P: paratype. AM, DQ: sequences deposited as AM296016 and DQ832701, respectively. **b** Pairwise genetic distances within and between *Xenoturbella* species calculated from *cox1* alignments. “‘shallow’ clade” and “‘deep’ clade” show inter-species differences between species belonging to each clade only. In “all species”, inter-species differences between all xenoturbellid species are shown. Each box represents the inner 50% quantile (median marked by the solid line). Whiskers extend to 1.5 times the interquartile range, with circles indicating outliers. Original data are available in Additional file 6: Table S1

Phylogenetic analyses using nucleotide alignments of the six reported mitochondrial genomes and those of the two new specimens showed that, in accordance with a previous study [45], *Xenoturbella* species are clustered into two major groups (Fig. 4a). *Xenoturbella japonica* is a sister group to a clade consisting of *X. bocki* and *X. hollandorum*, a group which was termed ‘shallow’ in Rouse et al. (2016) [45]. The other group, comprising *X. profunda*, *X. churro* and *X. monstrosa*, formed a clade that has previously been termed ‘deep’ [45]. All nodes in the tree were strongly (BP = 100) supported.

The pairwise genetic distances between the two collected specimens were compared with those of other xenoturbellids. Analyses using *cox1*, in which sequence data is available for the largest number of *Xenoturbella* specimens, showed that the genetic distance between the two new specimens are larger than almost all intra-species variations, but smaller than all inter-species variations (Fig. 4b, Additional file 6: Table S1). Analyses on either whole mitochondrial genomes, mitochondrial protein coding genes, or Histone H3 showed that the genetic distances between the two collected specimens were the smallest in all available pairwise genetic distance matrices (Additional file 7: Table S2).

Phylogenetic analyses of metazoans using mitochondrial genome sequences for the two new specimens resulted in different topology between data sets (Additional file 8: Figure S3, Additional file 9: Figure S4, Additional file 10: Figure S5 and Additional file 11: Figure S6). In agreement with previous studies [14, 45], this implies that mitochondrial genomes alone may not be suitable for resolving the phylogenetic positions of *Xenoturbella* and Xenacoelomorpha.

## Discussion

The two new specimens collected off the Japanese coast were confirmed as xenoturbellids based on their morphology (Figs. 1, 2 and 3) and molecular phylogenetic analyses (Fig. 4, Additional file 8: Figure S3, Additional file 9: Figure S4, Additional file 10: Figure S5 and Additional file 11: Figure S6). The latter also showed that they do not belong to the five known species of *Xenoturbella* (Fig. 4, Additional file 6: Table S1 and Additional file 7: Table S2). But are the two specimens different species? Analyses of pairwise genetic distances showed that the distance between the two new specimens is larger than all xenoturbellid intra-species variations, except for three outliers seen among *X. bocki cox1* sequences, but smaller than those of any inter-species pairs (Fig. 4b, Additional file 6: Table S1 and Additional file 7: Table S2). The two specimens were collected roughly 600 km apart, but *X. monstrosa* has been reported from two locations more than 2500 km apart, and *X. monstrosa* and *X. churro* have been shown to inhabit the same locality [45]. Therefore, the distance between the collection locations is not informative for species differentiation. There are morphological differences between the two specimens (Figs. 1, 2 and 3; Additional files 1: Video S1, Additional files 2: Video S2 and Additional files 3: Video S3), but these may be due to age differences. Therefore, until further specimens are collected, the two new xenoturbellids are considered as the same species, *X. japonica*, with the larger female specimen as the holotype and the smaller specimen, probably a juvenile, as a paratype.

DNA extraction experiments have yielded contaminating bivalve DNA from four of the five known *Xenoturbella* species [45, 50, 51]. In this study, DNA sequences with similarities to those of the following bivalves were detected from *X. japonica*; *Nucula nucleus*, *Ennucula cardara*, *Acila castrensis* and *Limaria fragilis*, further supporting the theory that *Xenoturbella* feeds on bivalves. Since none of these species were collected together with *X. japonica*, further studies are needed to verify if it feeds on these exact species, or on closely related species in the area.

All Pacific xenoturbellid species, including *X. japonica* in this study, were collected from depths greater than 500 m. The type locality for *X. bocki* is around 100 m, but the area is within the Gullmarsfjord, Sweden, known for its biodiversity of deep-sea animals living at a relatively shallow depth [11]. Therefore, *Xenoturbella* is probably a deep-sea fauna ancestrally. Considering Xenacoelomorpha, a clade inhabiting the deep-sea floor has been reported as a sister group to the rest of the acoels [52]. Comparison of acoels and *Xenoturbella* for morphological and physiological traits necessary for adapting to the deep-sea environment may provide insights into the ancestral habitat of Xenacoelomorpha.

We discovered a new structure in *Xenoturbella* - the frontal pore. It opens at the anterior tip of the animal, just below the side furrow, and connects with the ventral glandular network (Fig. 3, Additional file 1: Video S1, Additional file 2: Video S2 and Additional file 3: Video S3). Although previously not described from the well-studied *X. bocki*, reinvestigation revealed that the frontal pore is also present in this species (Fig. 3e–h, Additional file 1: Video S1 and Additional file 3: Video S3). MicroCT scanning, a method previously not applied to *Xenoturbella*, proved to be instrumental in this discovery. Since collections of *Xenoturbella* can be rare (less than four specimens each for 4 of the 6 species [45]), this non-destructive method promises to be a powerful tool for investigating the internal morphology of this soft-bodied animal. A frontal organ, which consists of a frontal pore at the anterior end and a group of mucous glands (frontal glands) connected to the pore, is present in a number of acoel and nemertodermatid species [53–56], and has been suggested to be an ancestral trait for Acoelomorpha [57, 58]. Similarities in their overall morphologies and position tempts us to speculate that the frontal pore and ventral glandular network of xenoturbellids and the frontal organ of acoelomorphs are homologous and that the structure is a synapomorphy for Xenacoelomorpha. However, there are differences in their structures, with the acoelomorph frontal organ having a more elaborate morphology [53–56]. Comparisons of the frontal pore and ventral glandular network of xenoturbellids and the acoelomorph frontal organ with respect to ultrastructure and the gene regulatory networks employed for morphogenesis may help to clarify the evolutionary relationship of these structures.

A previous study revealed that the five reported xenoturbellid species could be divided into two clades, ‘shallow’ and ‘deep’ [45]. Species of the first clade, *X. bocki* and *X. hollandorum*, live on the sea floor no deeper than 650 m and their body lengths are shorter than 4 cm. The ventral mouth is diamond shaped when animals are relaxed chemically. The ventral glandular network is not so conspicuous in this clade. The second clade consists of *X. monstrosa*, *X. churro* and *X. profunda*. These species have been reported from the seafloor between 1700 and 3700 m deep. They grow to be larger than the ‘shallow’ clade, reaching 10–20 cm in body length, with an obvious ventral glandular network and an oval mouth. Molecular phylogenetic analyses showed that *X. japonica* belong in the ‘shallow’ clade (Fig. 4a). The collected depth, body length, and the lack of an obvious ventral glandular network of the paratype specimen fit the characters of the ‘shallow’ clade. However, the holotype specimen shows characteristics of both clades. It was collected at 380–560 m depth, corresponding to the depth of the ‘shallow’ group. The body length of 5.3 cm falls between the two clades. Its oval mouth and conspicuous ventral glandular network suggest affinities to the ‘deep’ group. It is worth noting that the ventral epidermal network ends just anterior to the ring furrow in *X. japonica*, whereas the network passes the furrow posteriorly in *X. monstrosa*, *X. churro* and *X. profunda* [45]. Together with the lack of mature gametes, it is possible that the *X. japonica* holotype specimen is also still not fully grown, with the ventral epidermal network expanding posteriorly and the body lengthening with growth. Considering the phylogenetic position of *X. japonica*, it is parsimonious to regard characters common between this species and species in the ‘deep’ clade were present in the last common ancestor of *Xenoturbella*: body length over 5 cm, a well-developed ventral glandular network (and probably the frontal pore), and an oval mouth.

## Conclusions

We have reported here the collection of two new specimens of *Xenoturbella*. Our discovery of *Xenoturbella* from the western Pacific Ocean greatly broadens the biogeographical range of the animal (Additional file 4: Figure S1). Molecular phylogenetic analyses of the two specimens revealed that they do not belong to the five previously described species. We propose that the new specimens belong to the same species, *X. japonica*, with the holotype specimen being female and the paratype specimen as juvenile, until further specimens are collected. MicroCT scanning established the presence of the frontal pore and ventral glandular network in *Xenoturbella*, and we suggest these structures as a new synapomorphy for *Xenoturbella*. *X. japonica* shows traits of both the ‘shallow’ and ‘deep’ groups, and therefore will be an important species for research on *Xenoturbella* and Xenacoelomorpha. This species can be collected using a marine biological dredge within a 1 h boat trip, and so promises to be a valuable source of information for investigating the evolution and diversity of deuterostomes and bilaterians.

## Methods

### Collection and sample handling

The holotype specimen was collected as stated in the results. Pictures were taken using a Nikon Df fitted with AI AF Micro Nikkor 105 mm F2.8D or with a Leica DFC290 HD digital camera mounted on a Leica M205C stereomicroscope. 7% MgCl_2_ in freshwater was used to relax the animal. Pieces of the animal were dissected and fixed in RNAlater for DNA extraction and then the partially dissected specimen was fixed in 4% paraformaldehyde in filtered seawater overnight, washed, and kept in 70% ethanol.

Collection of the paratype specimen was performed as described in the results. Pictures of the live animal were taken with an OLYMPUS OM-D E-M5, fitted with OLYMPUS M. ZUIKO DIGITAL ED 60 mm F2.8 Macro lens using a pair of Morris Hikaru Komachi Di flashes. The animal was anaesthetized in MgCl_2_ solution isotonic to seawater and fixed in 10% neutral buffered formalin. The sample was stored in 10% neutral buffered formalin at room temperature for about 8 months. Pictures of the fixed specimen were taken with a Leica DFC290 HD digital camera mounted on a Leica M205C stereomicroscope. It was then transferred and preserved in 90% ethanol at 4 °C. Pieces of the animal were dissected and used for DNA extraction and then microCT scanning was performed on the partially dissected specimen.

Both specimens are deposited in the National Museum of Nature and Science, Tsukuba (NSMT), Japan.

*Xenoturbella bocki* was collected as previously described in [3, 11] at the Sven Lovén Centre for Marine Infrastructure, Gothenburg University, Sweden. Animals were fixed in 4% paraformaldehyde in filtered sea water at 4 °C overnight, dehydrated through a graded ethanol series and stored in 70% or 95% ethanol. Histological sections were made and photographed according to our previous studies [3, 33].

### MicroCT scanning and image analysis

Samples stored in 70% or 95% ethanol were either stained with 1% PTA solution in 70% ethanol [59] or were rehydrated through a graded ethanol series and stained with 25% Lugol solution (1:3 mixture of Lugol’s solution and deionized distilled water) [60]. The only exception was the paratype specimen, in which both staining methods were performed on a single sample. The staining time ranged from 3 to 48 h, depending on the size of the specimen. The stained samples were scanned using an X-ray microCT system (ScanXmate-E090S105; Comscan Techno) at a tube voltage peak of 60 kV and a tube current of 130 μA. During scanning, samples were rotated 360 degrees in steps of 0.1 to 0.18 degrees, generating 2000–3600 projection images of 992 × 992 pixels. Images were reconstructed using software provided with the X-ray microCT system (coneCTexpress; Comscan Techno) and volume data consisting of several hundred 8 bit TIFF files were obtained. For the *X. japonica* holotype and *X. bocki* No.2 samples, parts of the samples were scanned separately in order to obtain higher resolution images, and the reconstructed TIFF files were combined to generate data for the whole specimen. These data were used for analyses. 2D and 3D tomographic images were obtained using the OsiriX [61] and Tri/3D–BON (Ratoc System Engineering) software programs. Details of staining and scanning procedures are summarized in Additional file 12: Table S3.

### DNA extraction and sequencing

Genomic DNA was extracted from a piece of the *X. japonica* holotype specimen stored in RNAlater solution (Ambion) using DNeasy Blood & Tissue Kit (Qiagen). Cytochrome-*c*-oxidase subunit I (*cox1*) and cytochrome B (*cob*) fragments were amplified by PCR, and purified with QIA quick Gel extraction Kit (Qiagen). Sequencing was carried out by a DNA sequencing service (Fasmac). Specific primers were designed from the sequences of the *cox1* and *cob*, and the full-length mitochondrial genome was amplified as two overlapping fragments by PCR. Barcode sequences were added to 5′ end of the specific primers, and the PCR products were sequenced by Macrogen Japan using PacBio RS II Multiplexing Targeted Sequencing. PCR conditions (taq-polymerases, primers and amplification parameters) are shown in Additional file 13: Table S4. PacBio RS II reads were classified according to their barcode sequences using standalone BLAST (blastn, version 2.2.29), and aligned with MAFFT v7.221 [62] with a gap opening penalty of 0.1. Barcode sequences and ambiguous sites in the alignments were excluded using in-house Perl scripts. The mitochondrial genome of the *X. japonica* holotype specimen was obtained by concatenating two fragments, assembled from 573 reads (Average Coverage: 428) and 743 reads (Average Coverage: 558), respectively, from PacBio library.

Genomic DNA of the *X. japonica* paratype specimen was extracted using Allprep DNA/RNA FFPE Kit (QIAGEN). The extracted genomic DNA was fragmented to a target length of 600 bp by S220 Focused-Ultrasonicator (Covaris). Before preparing the library, the fragmented DNA was repaired using PreCR Repair Mix (New England Biolabs). Using the repaired DNA, a paired-end sequencing library was prepared with KAPA Hyper Prep Kit (KAPA Biosystems) according to the manufacturer’s instructions. *Xenoturbella japonica* paratype specimen genome sequence data were obtained using a 300 bp paired-end protocol on an Illumina Miseq instrument (Illumina). BLAST searches against mitochondrial sequences registered in Refseq and Silva SSU 119 were performed to extract contigs with similarities to mitochondrial genomes and ribosomal small subunit RNAs, respectively. The mitochondrial genome of the paratype was reconstructed by assembling 40,879 reads from a MiSeq library (Average Coverage: 465) using MITObim v1.8 [63], the *X. bocki* mitochondrial genome as an initial reference sequence.

The mitochondrial genomes of both *X. japonica* specimens were annotated using the MITOS web server, employing translation table 5 [64].

The nucleotide sequence of Histone H3 from the *X. japonica* holotype specimen was amplified by PCR, purified with QIA quick Gel extraction Kit (Qiagen), and ligated into pMD20-T vector (Takara Bio). The nucleotide sequences of the vectors were directly amplified from clones by PCR, purified using Exonuclease I and Calf intestine alkaline phosphatase (both Takara Bio), and sequenced using a DNA sequencing service (Fasmac). The PCR conditions (taq-polymerases, primers and amplification parameters) are shown in Additional file 13: Table S4. The nucleotide sequence of the paratype specimen Histone H3 was reconstructed from reads of MiSeq library: 182 reads showing high similarity (>85% identity) to the nucleotide sequence of *X. bocki* Histone H3 were identified in MiSeq library using a standalone BLASTn search and were aligned using MAFFT v7.221 with gap opening penalty of 0.5.

Sequences obtained were deposited in the International Nucleotide Sequence Database (INSD) through the DNA Data Bank of Japan (DDBJ).

### Molecular phylogenetic analyses

For phylogenetic analyses of xenoturbellids, nucleotide sequences of the eight available mitochondrial genomes were aligned using MAFFT L-INS-i, and refined by Gblocks using the following parameters: b2 = 75%, b3 = 5, b4 = 5, b5 = half [65]. Phylogenetic relationships were inferred using RAxML (v8.1.11) under the GTR + gamma model with 100 bootstrap replicates.

For calculating pairwise genetic distances, amino acid sequences of 13 concatenated mitochondrial protein-coding genes and nucleotide sequences of whole mitochondrial genomes, nuclear histone H3 and *cox1* genes were aligned using MAFFT L-INS-i. Pairwise distances were calculated using the R package ‘phangorn’ [66], employing the MtMam model for amino acid sequences and the JC69 model for nucleotide sequences. A nuclear histone H3 sequence was not available for *X. churro*. Among *cox1* alignments, genetic distances between two non-overlapping sequences were not calculated.

Four different phylogenetic analyses of bilaterian mitochondrial genomes were performed. In three analyses (*i*–*iii*), each dataset was aligned using MAFFT L-INS-i and ambiguous sites were removed with Gblocks using stringent parameters (b2 = 75%, b3 = 5, b4 = 5, b5 = half) [65]. RAxML was run under the LG4X + gamma model with the data partitioned by genes. In a fourth analysis (*iv*), genomes were aligned with MAFFT FFT-NS-i (faster but less accurate than MAFFT L-INS-i) and refined by Gblocks 0.91b [67] using less stringent condition parameters (b2 = 65%, b3 = 10, b4 = 5, b5 = all) [68]. The maximum likelihood (ML) analysis was carried out with RAxML v8.1.1 [69] under the GTR + gamma model with the data partitioned (*−q* option) by genes. Datasets used in the four analyses were amino acid sequences of 13 mitochondrial protein-coding genes (*atp6* and *8*, *cob*, *cox1–3*, *nad1–6* and *nad4L*) from 31 metazoans and six *Xenoturbella* sequences (same dataset as ref. [45]) with the addition of two new sequences acquired in this study, with the following differences between analyses. (*i*) all four acoelomorph species were excluded; (*ii*) all 31 metazoans and eight *Xenoturbella* data were used; (*iii*) non-bilaterian metazoans (3 sponges and 3 cnidarians) were excluded; (*iv*) all eight *Xenoturbella* and 31 metazoans mitochondrial genomes were used. The alignment lengths of the four phylogenetic analyses were: (*i*) 2179 aa; (*ii*) 2208 aa; (*iii*) 2202 aa; (*iv*) 2791 aa.

## Additional files

Additional file 1: Video S1. Volume rendering image reconstructed from micoCT scans showing external morphology of *X. japonica* holotype (H), paratype (P) and *X. bocki.* (MP4 19263 kb)

Additional file 2: Video S2. MicroCT sections showing internal structures of *X. japonica* holotype female*.* vgn, ventral glandular network. (MP4 19089 kb)

Additional file 3: Video S3. MicroCT sections showing internal structures of *X. japonica* paratype juvenile and *X. bocki.* vgn, ventral glandular network. (MP4 19407 kb)

Additional file 4: Figure S1. Distribution of *Xenoturbella*. a: Collection sites of the two specimens of *X. japonica* from the western Pacific. H: holotype, P: paratype. b: Worldwide distribution of *Xenoturbella*. Only sites where the species of the collected specimens were confirmed by molecular phylogenetic analyses are shown. The map and plots were generated with GMT5 software [71]. *Xb*: *X. bocki*, *Xc*: *X. churro*, *Xh*: *X. hollandorum*, *Xj*: *X. japonica* sp. nov., *Xm*: *X. monstrosa*, *Xp*: *X. profunda.* (PDF 1477 kb)

Additional file 5: Figure S2. Linearized mitochondrial genome maps of *X. japonica* sp. nov. holotype, paratype and *X. bocki.* Red; protein coding genes, blue; tRNA, green; rRNA. Gene orders of both *X. japonica* specimens were identical with that of *X. bocki*. (PDF 904 kb)

Additional file 6: Table S1. Pairwise genetic distances of nucleotide *cox1* alignments. Intra-species genetic distances of *X. japonica*, *X. bocki*, *X. profunda* and *X. monstrosa* are colored red, light green, light blue and light purple, respectively. Inter-species genetic distances are shown in gray. Inter-species genetic distances between species in the ‘shallow’ clade are surrounded by an orange square, and those in the ‘deep’ clade are surrounded by cobalt squares. (PDF 74 kb)

Additional file 7: Table S2. Pairwise genetic distances of *Xenoturbella* species. (PDF 52 kb)

Additional file 8: Figure S3. Maximum likelihood tree of metazoans (excluding Acoelomorpha) based on 13 mitochondrial protein-coding genes. Bootstrap values are shown at the nodes. Bilaterian taxon names are indicated to the right of the tree. H: holotype, P: paratype. AM, DQ: sequences deposited as AM296016 and DQ832701, respectively. (PDF 1200 kb)

Additional file 9: Figure S4. Maximum likelihood tree of metazoans based on 13 mitochondrial protein-coding genes. Bootstrap values are shown at the nodes. Bilaterian taxon names are indicated to the right of the tree. H: holotype, P: paratype. AM, DQ: sequences deposited as AM296016 and DQ832701, respectively. (PDF 1249 kb)

Additional file 10: Figure S5. Unrooted maximum likelihood tree of bilaterians based on 13 mitochondrial protein-coding genes. Bootstrap values are shown at the nodes. Bilaterian taxon names are marked with dashed lines. H: holotype, P: paratype. AM, DQ: sequences deposited as AM296016 and DQ832701, respectively. (PDF 920 kb)

Additional file 11: Figure S6. Maximum likelihood tree of metazoans based on 13 mitochondrial protein-coding genes with less stringent conditions. Bootstrap values are shown at the nodes. Bilaterian taxon names are indicated to the right of the tree. H: holotype, P: paratype. AM, DQ: sequences deposited as AM296016 and DQ832701, respectively. (PDF 1458 kb)

Additional file 12: Table S3. Details for microCT scans. (PDF 52 kb)

Additional file 13: Table S4. Primers list and PCR conditions. (PDF 57 kb)

## Abbreviations

Cob: Cytochrome B
cox1: Cytochrome C oxidase subunit I
DDBJ: DNA Data Bank of Japan
FRV: Fisheries research vessel
INSD: International Nucleotide Sequence Database
JAMBIO: Japanese Association for Marine Biology
microCT: micro computed tomography
NSMT: National Museum of Nature and Science, Tsukuba, Japan
ROVs: Remotely operated vehicles
rSSU: Ribosomal small subunit
RV: Research vessel
