# Correction to: A new species of *Xenoturbella* from the western Pacific Ocean and the evolution of *Xenoturbella*

**DOI:** 10.1186/s12862-018-1190-5

**Published:** 2018-06-07

**Authors:** Hiroaki Nakano, Hideyuki Miyazawa, Akiteru Maeno, Toshihiko Shiroishi, Keiichi Kakui, Ryo Koyanagi, Miyuki Kanda, Noriyuki Satoh, Akihito Omori, Hisanori Kohtsuka

**Affiliations:** 1 Shimoda Marine Research Center, University of Tsukuba, 5-10-1, Shimoda, Shizuoka, 415-0025 Japan; 2 Mammalian Genetics Laboratory, National Institute of Genetics, 1111 Yata, Mishima, Shizuoka, 411-8540 Japan; 3 Faculty of Science, Hokkaido University, N10 W8, Kita-ku, Sapporo, Hokkaido 060-0810 Japan; 4 DNA Sequencing Section, Okinawa Institute of Science and Technology Graduate University, Onna, Okinawa 904-0495 Japan; 5 Marine Genomics Unit, Okinawa Institute of Science and Technology Graduate University, Onna, Okinawa 904-0495 Japan; 6 Misaki Marine Biological Station, The University of Tokyo, 1024 Koajiro, Misaki, Miura, Kanagawa 238-0225 Japan; 7 Present address: Sado Marine Biological Station, Faculty of Science, Niigata University, Sado, Niigata 952-2135 Japan

## Correction

After publication of Nakano et al. (2017) [1], the authors became aware of the fact that the new species-group name erected for the two specimens of a Japanese xenoturbellid species in the article is not available because Nakano et al. (2017) [1] does not meet the requirement of the amendment of Article 8.5.3 of the International Code of Zoological Nomenclature (the Code) [2]. The authors therefore describe the two xenoturbellids as a new species again in this correction article. Methods for morphological observation, DNA extraction and sequencing were as described in Nakano et al. (2017) [1]. The holotype and paratype specimens are deposited in the National Museum of Nature and Science, Tsukuba (NSMT), Japan. The DNA sequences obtained were deposited in the International Nucleotide Sequence Database (INSD).

**Genus** ***Xenoturbella*** **Westblad, 1949** [3]

***Xenoturbella japonica*** **sp. nov.**

http://zoobank.org/6C4EA6F8-8AC1-4511-A59B-BCB60729A85A

(Figs. 1–3, Additional files 1–3 in Nakano et al. (2017) [1])

**Etymology.** Named for the locality where the specimens were collected.

**Holotype**. NSMT-Xe 2, female (Figs. 1, 3, Additional files 1, 2 in Nakano et al. (2017) [1]), off Jogashima, Miura, Kanagawa, Japan, 35°06.93″ N 139°33.72″ E to 35°06.95″ N 139°33.33″ E, 380–554 m depth, December 9th, 2015.

**Paratype.** NSMT-Xe 1, juvenile, sex unknown (Figs. 2, 3, Additional files 1, 3 in Nakano et al. (2017) [1]), Sanriku coast, Iwate, Japan, 39°37.86″ N 142°18.22″ E to 39°37.00″ N 142°17.60″ E, 517–560 m depth, July 18th, 2013.

**Description of female.** Based on holotype. Body 5.3 cm in length; pale orange with coloration getting darker toward the anterior. In live specimens, muscles hold the dorsal body wall in a W-shape (three ridges and two troughs). Body shape actively changes by contracting and elongating when alive. Ring furrow and side furrow are present. Ventral mouth present, oval-shaped, just anterior to ring furrow. Glandular network present over ventral surface, starting near anterior tip of body and ending just in front of ring furrow. Internally, body wall with epidermis, circular and longitudinal muscles, parenchyma and gastrodermis present. Oocytes present within intestine. Statocyst situated near anterior tip of body, just inside side furrow.

**Description of juvenile.** Based on paratype. Similar to female, but differs as follows: body 1.1 cm in length; pale orange in color; dorsal body surface in live specimen smooth, lacking longitudinal ridges and troughs, similar to that of *X. bocki*; gametes not observed. Ventral glandular network not detected externally, but observed with microCT imaging.

**Genetic information.** Whole mitochondrial genome sequences (15,244 bp in holotype; 15,249 bp in paratype) and partial Histone H3 gene sequences (346 bp in holotype; 413 bp in paratype) were determined and deposited as INSD accession numbers LC228486, LC228485, LC228579 and LC228578, respectively.

**Remarks.** The same species name presented in Nakano et al. (2017) [1] is nomen nudum as the publication does not meet the requirement of the amendment of Article 8.5.3 of the Code [2]. The present erratum fully meets the amended provisions of the Code, and thus this work makes the specific name *japonica* in the combination of *Xenoturbella* japonica available.

**Acknowledgements**

The authors would like to thank Takafumi Nakano for discussions concerning this erratum.
