# Supplementary material for: A new species of Xenoturbella from the western Pacific Ocean and the evolution of Xenoturbella
Source: BMC Evol Biol. 2017 Dec 18;17:245. doi: 10.1186/s12862-017-1080-2 (PMC5733810; doi:10.1186/s12862-017-1080-2)
Supplement: Supplementary file 4 — Distribution of Xenoturbella. a: Collection sites of the two specimens of X. japonica from the western Pacific. H: holotype, P: paratype. b: Worldwide distribution of Xenoturbella. Only sites where the species of the collected specimens were confirmed by molecular phylogenetic analyses are shown. The map and plots were generated with GMT5 software [71]. Xb: X. bocki, Xc: X. churro, Xh: X. hollandorum, Xj: X. japonica sp. nov., Xm: X. monstrosa, Xp: X. profunda. (PDF 1477 kb) [file 12862_2017_1080_MOESM4_ESM.pdf]

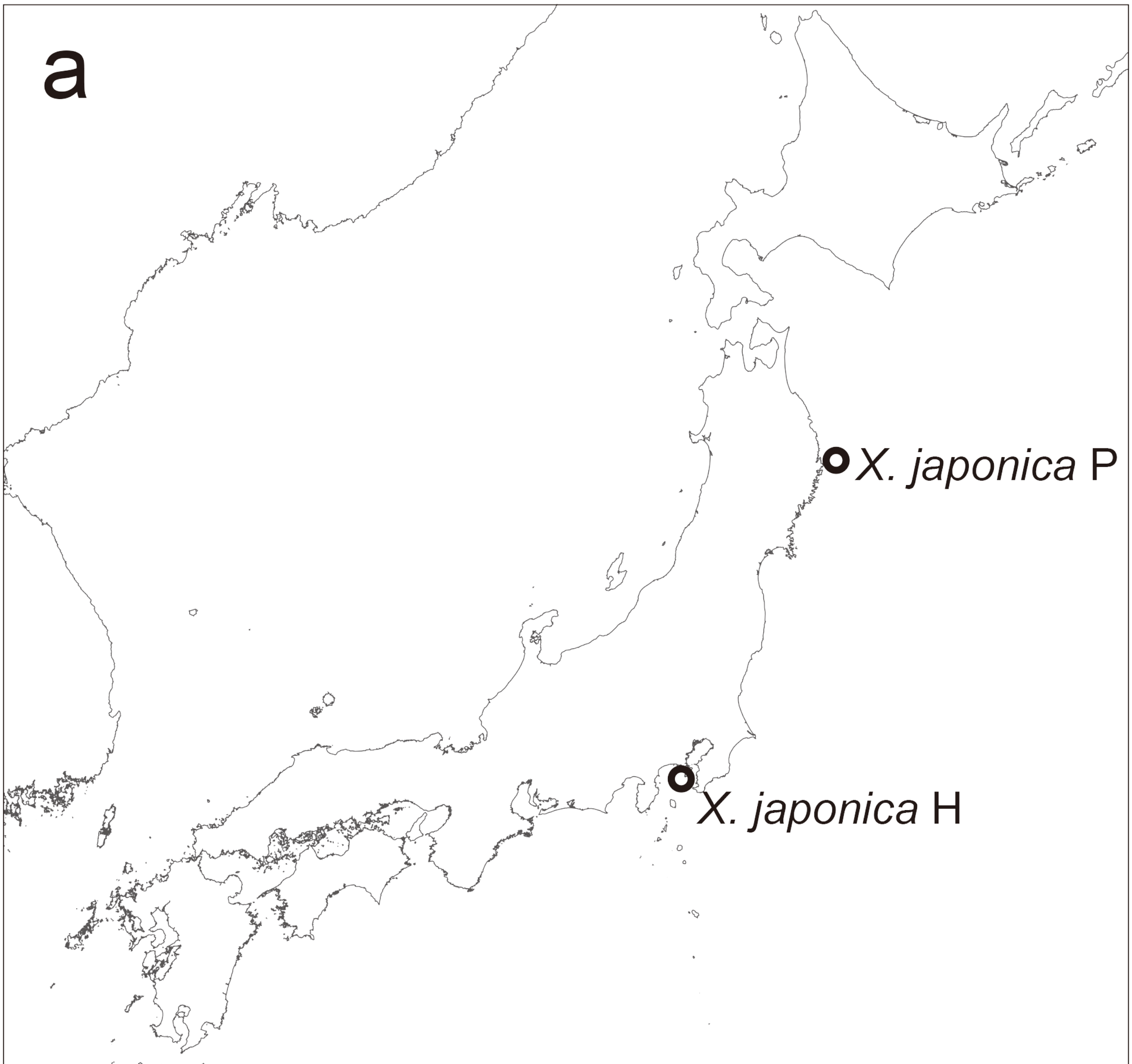

Additional file 4: Figure S1. Distribution of *Xenoturbella*. a: Collection sites of the two specimens of *X. japonica* from the western Pacific. H: holotype, P: paratype. b: Worldwide distribution of *Xenoturbella*. Only sites where the species of the collected specimens were confirmed by molecular phylogenetic analyses are shown. The map and plots were generated with GMT5 software [71]. *Xb*: *X. bocki*, *Xc*: *X. churro*, *Xh*: *X. hollandorum*, *Xj*: *X. japonica* sp. nov., *Xm*: *X. monstrosa*, *Xp*: *X. profunda*.
