# Supplementary material for: A new species of Xenoturbella from the western Pacific Ocean and the evolution of Xenoturbella
Source: BMC Evol Biol. 2017 Dec 18;17:245. doi: 10.1186/s12862-017-1080-2 (PMC5733810; doi:10.1186/s12862-017-1080-2)
Supplement: Supplementary file 5 — Linearized mitochondrial genome maps of X. japonica sp. nov. holotype, paratype and X. bocki. Red; protein coding genes, blue; tRNA, green; rRNA. Gene orders of both X. japonica specimens were identical with that of X. bocki. (PDF 904 kb) [file 12862_2017_1080_MOESM5_ESM.pdf]

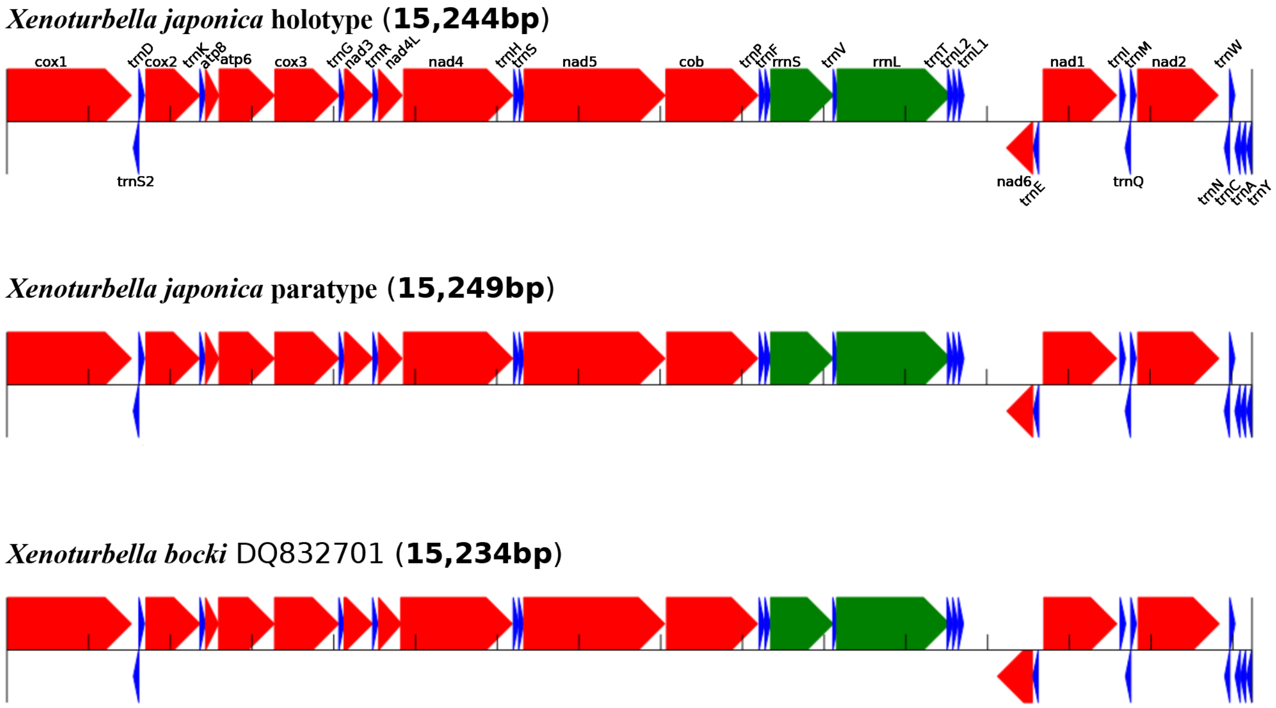

Additional file 5: Figure S2. Linearized mitochondrial genome maps of *X. japonica* sp. nov. holotype, paratype and *X. bocki*. Red; protein coding genes, blue; tRNA, green; rRNA. Gene orders of both *X. japonica* specimens were identical with that of *X. bocki*.
