# Supplementary material for: A new species of Xenoturbella from the western Pacific Ocean and the evolution of Xenoturbella
Source: BMC Evol Biol. 2017 Dec 18;17:245. doi: 10.1186/s12862-017-1080-2 (PMC5733810; doi:10.1186/s12862-017-1080-2)
Supplement: Supplementary file 6 — Pairwise genetic distances of nucleotide cox1 alignments. Intra-species genetic distances of X. japonica, X. bocki, X. profunda and X. monstrosa are colored red, light green, light blue and light purple, respectively. Inter-species genetic distances are shown in gray. Inter-species genetic distances between species in the ‘shallow’ clade are surrounded by an orange square, and those in the ‘deep’ clade are surrounded by cobalt squares. (PDF 74 kb) [file 12862_2017_1080_MOESM6_ESM.pdf]

Additional file 6: Table S1. Pairwise genetic distances of nucleotide *cox1* alignments. Intra-species genetic distances of *X. japonica*, *X. bocki*, *X. profunda* and *X. monstrosa* are colored red, light green, light blue and light purple, respectively. Inter-species genetic distances are shown in gray. Inter-species genetic distances between species in the 'shallow' clade are surrounded by an orange square, and those in the 'deep' clade are surrounded by cobalt squares.

	Accession Number	*X. japonica*		*X. hollandorum*	*X. churro*	*X. bocki*											*X. profunda*							*X. monstrosa*		
		LC228486	LC228485	KT862787	KT862786	AM296016	AY291293	AY461796	AY461797	AY461798	DQ832701	JX277096	AY461792	AY461793	AY461794	AY461795	KT884816	KT884817	KT884818	KT884820	KT884822	KT884824	KT884826	KU232613	KU232614	KT862788
X. japonica	LC228486	-																								
	LC228485	0.03234	-																							
X. hollandorum	KT862787	0.18558	0.18475	-																						
X. churro	KT862786	0.21040	0.21383	0.23297	-																					
X. bocki	AM296016	0.17868	0.17704	0.06971	0.21691	-																				
	AY291293	0.18449	0.18279	0.06992	0.22251	0.00200	-																			
	AY461796	0.17688	0.17688	0.08554	0.21887	0.00498	0.00623	-																		
	AY461797	0.17980	0.17781	0.07780	0.21237	0.00472	0.00630	0.00788	-																	
	AY461798	0.17737	0.17737	0.07960	0.21850	0.00122	0.00243	0.00623	0.00472	-																
	DQ832701	0.18115	0.17950	0.06829	0.21863	0.00323	0.00133	0.00749	0.00789	0.00488	-															
	JX277096	0.20428	0.19747	0.06728	0.21115	NA	0.00523	0.01230	NA	NA	0.00698	-														
	AY461792	0.20978	0.20409	0.07586	0.21744	0.01309	0.00742	0.04721	0.04028	0.04028	0.01016	0.00698	-													
	AY461793	0.20617	0.20054	0.08325	0.21755	0.01009	0.00894	0.02014	0.01336	0.01336	0.01738	0.00174	0.00726	-												
	AY461794	0.17326	0.17326	0.07393	0.21033	0.00160	0.00320	0.00480	0.00641	0.00160	0.00481	NA	0.04028	0.01336	-											
	AY461795	0.17584	0.17584	0.07470	0.22054	0.00115	NA	0.00623	0.00630	0.00243	0.00229	NA	0.03341	0.02014	0.00320	-										
X. profunda	KT884816	0.21016	0.21016	0.23022	0.09691	0.20078	0.20131	0.18849	0.17996	0.17996	0.20065	0.20200	0.20244	0.20436	0.17996	0.17154	-									
	KT884817	0.21796	0.21016	0.23022	0.10025	0.20078	0.19937	0.18849	0.17996	0.17996	0.20065	0.19973	0.19480	0.19670	0.17996	0.17154	0.00885	-								
	KT884818	0.21916	0.21113	0.23385	0.09988	0.20146	0.20002	0.19012	0.18035	0.18035	0.20133	0.19973	0.19923	0.20120	0.18035	0.17070	0.00606	NA	-							
	KT884820	0.21796	0.21016	0.23022	0.10025	0.20078	0.19937	0.18849	0.17996	0.17996	0.20065	0.19973	0.19480	0.19670	0.17996	0.17154	0.00885	NA	NA	-						
	KT884822	0.21871	0.21088	0.23101	0.10057	0.20145	0.20005	0.19139	0.18272	0.18272	0.20133	0.19973	0.19545	0.19736	0.18272	0.17415	0.00888	NA	NA	NA	-					
	KT884824	0.21908	0.21123	0.23342	0.10073	0.20373	0.20235	0.19287	0.18413	0.18413	0.20360	0.20200	0.19769	0.19961	0.18413	0.17548	0.01188	0.00295	0.00302	0.00295	0.00295	-				
	KT884826	0.24652	0.22987	0.24150	0.11553	0.20592	0.20556	0.21068	0.19728	0.19728	0.20822	0.20721	0.20822	0.20822	0.19728	0.18413	0.00812	0.00202	0.00202	0.00202	0.00202	0.00609	-			
X. monstrosa	KU232613	0.18628	0.19693	0.20009	0.11811	0.18926	0.19425	NA	NA	NA	0.19425	0.18581	0.19425	0.18893	NA	NA	0.11090	0.11811	0.11811	0.11811	0.11811	0.12295	0.13148	-		
	KU232614	0.18628	0.19693	0.20009	0.12053	0.18926	0.19425	NA	NA	NA	0.19425	0.18581	0.19425	0.18893	NA	NA	0.10852	0.11570	0.11570	0.11570	0.11570	0.12053	0.12850	0.00206	-	
	KT862788	0.19516	0.20105	0.21469	0.11548	0.20579	0.21095	0.21225	0.21030	0.21364	0.20579	0.18849	0.19843	0.19867	0.21245	0.21595	0.10530	0.11208	0.11379	0.11208	0.11243	0.11604	0.12980	0.01038	0.01247	-
