# Supplementary material for: A new species of Xenoturbella from the western Pacific Ocean and the evolution of Xenoturbella
Source: BMC Evol Biol. 2017 Dec 18;17:245. doi: 10.1186/s12862-017-1080-2 (PMC5733810; doi:10.1186/s12862-017-1080-2)
Supplement: Supplementary file 7 — Pairwise genetic distances of Xenoturbella species. (PDF 52 kb) [file 12862_2017_1080_MOESM7_ESM.pdf]

Additional file 7: Table S2. Pairwise genetic distances of *Xenoturbella* species.

a. Pairwise genetic distances of mitochondrial genomes. The nucleotide alignments of whole mitochondrial genomes are shown below diagonal and the amino acid alignments of 13 protein-coding genes above. AM296016 was used for *X. bocki*.

	X. bocki	*X. hollandorum*	*X. churro*	*X. monstrosa*	*X. profunda*	*X. japonica* H	*X. japonica* P
X. bocki	-	0.02663595	0.24558476	0.23701181	0.25129119	0.0999591	0.10139707
X. hollandorum	0.06746209	-	0.25352857	0.24489155	0.25638923	0.10155309	0.10218576
X. churro	0.28173372	0.28359769	-	0.05779161	0.06109594	0.24908236	0.2472855
X. monstrosa	0.27331117	0.27607394	0.12358723	-	0.06360262	0.24170651	0.23836329
X. profunda	0.27648103	0.27552195	0.10328519	0.11609216	-	0.25843539	0.25657251
X. japonica H	0.1901699	0.18878925	0.28260853	0.27636805	0.28226841	-	0.01484132
X. japonica P	0.19155196	0.18906777	0.28279789	0.27598452	0.28207252	0.03073818	-

b. Pairwise genetic distances of nucleotide alignments of nuclear Histone H3. FJ555303 was used for *X. bocki*.

	X. bocki	*X. hollandorum*	*X. churro*	*X. monstrosa*	*X. profunda*	*X. japonica* H	*X. japonica* P
X. bocki	-						
X. hollandorum	0.03360562	-					
X. churro	?	?	-				
X. monstrosa	0.1261294	0.15600043	?	-			
X. profunda	0.13043458	0.15024224	?	0.01941856	-		
X. japonica H	0.28316165	0.22244223	?	0.27472938	0.26556896	-	
X. japonica P	0.28316165	0.22244223	?	0.28400306	0.27472938	0.01769992	-
