# Supplementary material for: A new species of Xenoturbella from the western Pacific Ocean and the evolution of Xenoturbella
Source: BMC Evol Biol. 2017 Dec 18;17:245. doi: 10.1186/s12862-017-1080-2 (PMC5733810; doi:10.1186/s12862-017-1080-2)
Supplement: Supplementary file 12 — Details for microCT scans. (PDF 52 kb) [file 12862_2017_1080_MOESM12_ESM.pdf]

Additional file 12: Table S3. Details for microCT scans

Object	Stained solution	Stained Time	Scanned part	Frame average	Rotation Steps	Number of projections	Voxel Size (μm)*	Number of scanned parts	Dataset size (GB)**	Figure	Video	Data File Name***
X. japonica holotype female	25% Lugol	2days	whole body	2	0.18°	2000	14.50	3	1.75	Fig. 1d	Videos S1, S2	1_X_jp_halotype_L1
			anterior	2	0.18°	2000	5.20	1	0.72	Fig. 1e,f		2_X_jp_halotype_L2
			anterior	2	0.10°	3600	4.89	3	2.32	Fig. 3a,b	Video S2	3_X_jp_halotype_L3
X. japonica paratype juvenile	25% Lugol	6hr30min	whole body	2	0.18°	2000	5.41	1	0.62	Fig. 2d,e	Videos S1, S3	4_X_jp_paratype_L1
			anterior	2	0.18°	2000	3.73	1	0.84			
	1% PTA	1day	whole body	2	0.18°	2000	5.41	1	0.51			
			anterior	2	0.18°	2000	3.73	1	0.83			
			anterior	2	0.18°	2000	3.51	1	0.74	Figs. 2c, 3c,d		5_X_jp_paratype_P1
X. bocki sample 1	25% Lugol	3hrs	whole body	4	0.10°	3600	3.20	1	0.62			
X. bocki sample 2	0.3% PTA	2days	whole body	2	0.10°	3600	3.22	2	0.75		Videos S1, S3	6_X_bocki_P1
			anterior	4	0.10°	3600	3.22	1	0.49	Fig. 3e,f		7_X_bocki_P2

* : Isotropic voxel resolution

**: Total size (gigabyte) of 8bit TIFF format image files

***: File Name in figshare repository [70]
