# Supplementary material for: A new species of Xenoturbella from the western Pacific Ocean and the evolution of Xenoturbella
Source: BMC Evol Biol. 2017 Dec 18;17:245. doi: 10.1186/s12862-017-1080-2 (PMC5733810; doi:10.1186/s12862-017-1080-2)
Supplement: Supplementary file 13 — Primers list and PCR conditions. (PDF 57 kb) [file 12862_2017_1080_MOESM13_ESM.pdf]

Additional file 13: Table S4. Primers list and PCR conditions

Gene, Region	Forward Primer	sequence (5'-3')	Reverse Primer	sequence (5'-3')	Citation	Taq	Cycling Condition
cob	XenoCOB_F1	CGA AAA ACM CAC CCM BTA ATH AAA AT	XenoCOB_R1	CCR TTY ATR TGD ANT GTT CGT ATT A	this study	TaKaRa Ex Taq (Takara Bio Inc.)	95˚C for 3 min. 40 cycles of 95˚C for 30 sec, 53˚C for 45 sec, 72˚C for 45 sec. Final extension of 72˚C for 5 min.
coxI	LCO1490	GGT CAA CAA ATC ATA AAG ATA TTG G	HCO2100	TAA ACT TCA GGG TGA CCA AAA AAT CA	Folmer et al. 1994	TaKaRa Ex Taq (Takara Bio Inc.)	94˚C for 3 min. 5 cycles of 94˚C for 30 sec, 47˚C for 45 sec, 72˚C for 1 min. 35 cycles of 94˚C for 30 sec, 52˚C for 45 sec, 72˚C for 1 min. Final extension of 72˚C for 5 min.
coxI-cob	Xsp1_cox1LF1	AGA GAT GTG TGA TGA CTA GGT ACA GGA CTA AGC ATA ATC ATT CG	Xsp1_cobLR1	GTC ATC ACA CAT CTC TGA TAT GTG AGA TTG AGT CAA AGG CTA T	this study	TaKaRa LA Taq (Takara Bio Inc.)	94˚C for 1 min. 35 cycles of 98˚C for 10 sec, 47˚C for 30 sec, 68˚C for 8 min. Final extension of 72˚C for 8 min.
cob-coxI	Xsp1_cobLF1	TAC GAC TAC ATA TCA GAT CGA TCT TCC ATC ACC TAG TAA TAT CT	Xsp1_cox1LR1	CTG ATA TGT AGT CGT ATA TTA GGG GTA GTA ATC AGT TTC CGA AG	this study	TaKaRa LA Taq (Takara Bio Inc.)	94˚C for 1 min. 35 cycles of 98˚C for 10 sec, 47˚C for 30 sec, 68˚C for 8 min. Final extension of 72˚C for 8 min.
Histone H3	HH3_31F	RTG GAT GGC GCA CAA GTT TG	HH3_373R	AAT GGC CAG GAC AAA GCA GA	Rouse et al. 2016	TaKaRa Ex Taq (Takara Bio Inc.)	95˚C for 3 min. 40 cycles of 95˚C for 30 sec, 53˚C for 45 sec, 72˚C for 45 sec. Final extension of 72˚C for 5 min.
pMD20-T Vector	M13RV	CAG GAA ACA GCT ATG AC	M13M4	GTT TTC CCA GTC ACG AC	this study	SapphireAmp Fast PCR Master Mix (Takara Bio, Japan)	94˚C for 1 min. 30 cycles of 98˚C for 5 sec, 50˚C for 5 sec, 72˚C for 20 sec. Final extension of 72˚C for 40 sec.
