# Supplementary material for: A new species of Xenoturbella from the western Pacific Ocean and the evolution of Xenoturbella
Source: BMC Evol Biol. 2017 Dec 18;17:245. doi: 10.1186/s12862-017-1080-2 (PMC5733810; doi:10.1186/s12862-017-1080-2)
Supplement: Supplementary file 10 — Unrooted maximum likelihood tree of bilaterians based on 13 mitochondrial protein-coding genes. Bootstrap values are shown at the nodes. Bilaterian taxon names are marked with dashed lines. H: holotype, P: paratype. AM, DQ: sequences deposited as AM296016 and DQ832701, respectively. (PDF 920 kb) [file 12862_2017_1080_MOESM10_ESM.pdf]

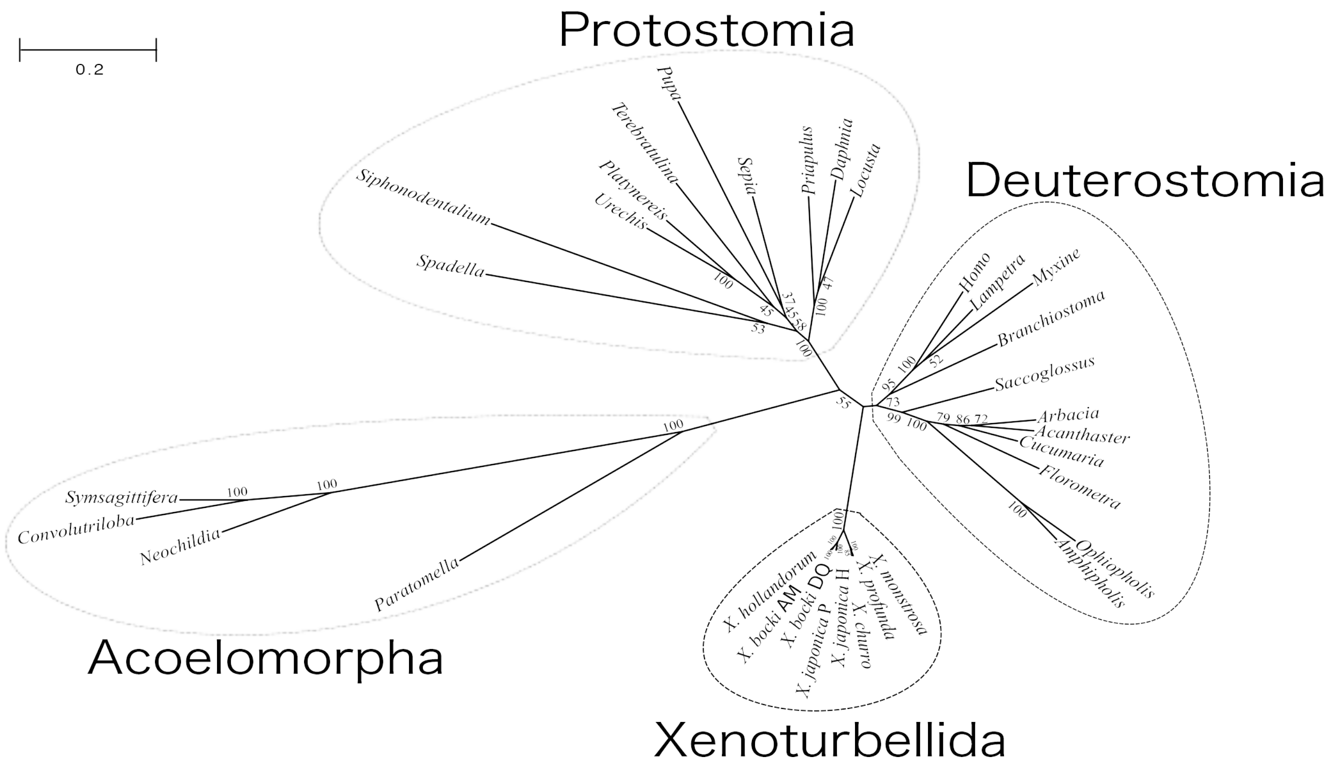

Additional file 10: Figure S5. Unrooted maximum likelihood tree of bilaterians based on 13 mitochondrial protein-coding genes. Bootstrap values are shown at the nodes. Bilaterian taxon names are marked with dashed lines. H: holotype, P: paratype. AM, DQ: sequences deposited as AM296016 and DQ832701, respectively.
